# BCL10 – Bridging CARDs to Immune Activation

**DOI:** 10.3389/fimmu.2018.01539

**Published:** 2018-07-04

**Authors:** Torben Gehring, Thomas Seeholzer, Daniel Krappmann

**Affiliations:** Research Unit Cellular Signal Integration, Institute of Molecular Toxicology and Pharmacology, Helmholtz Zentrum München – German Research Center for Environmental Health, Neuherberg, Germany

**Keywords:** adaptive immunity, T cell signaling, innate immunity, NF-kappa B, B-cell lymphoma/leukemia 10, CBM complex

## Abstract

Since the B-cell lymphoma/leukemia 10 (BCL10) protein was first described in 1999, numerous studies have elucidated its key functions in channeling adaptive and innate immune signaling downstream of CARMA/caspase-recruitment domain (CARD) scaffold proteins. While T and B cell antigen receptor (TCR/BCR) signaling induces the recruitment of BCL10 bound to mucosa-associated lymphoid tissue (MALT)1 to the lymphocyte-specific CARMA1/CARD11–BCL10–MALT1 (CBM-1) signalosome, alternative CBM complexes utilize different CARMA/CARD scaffolds in distinct innate or inflammatory pathways. BCL10 constitutes the smallest subunit in all CBM signalosomes, containing a 233 amino acid coding for N-terminal CARD as well as a C-terminal Ser/Thr-rich region. BCL10 forms filaments, thereby aggregating into higher-order clusters that mediate and amplify stimulation-induced signals, ultimately leading to MALT1 protease activation and canonical NF-κB and JNK signaling. BCL10 additionally undergoes extensive post-translational regulation involving phosphorylation, ubiquitination, MALT1-catalyzed cleavage, and degradation. Through these feedback and feed-forward events, BCL10 integrates positive and negative regulatory processes that govern the function as well as the dynamic assembly, disassembly, and destruction of CBM complexes. Thus, BCL10 is a critical regulator for activation as well as termination of immune cell signaling, revealing that its role extends far beyond that of a mere linking factor in CBM complexes.

## Discovery of BCL10 as an Adaptor for Card-Dependent NF-κB Activation

**Figure 1:**
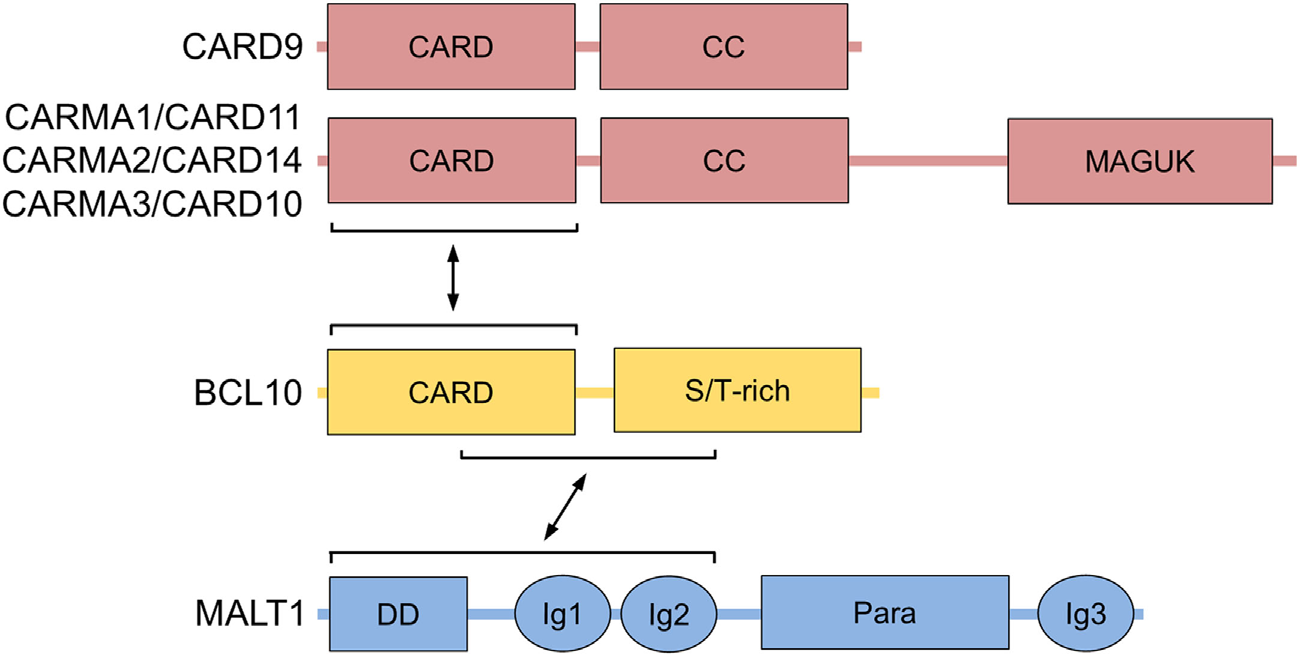
BCL10 structure and interactors. Domain structure of BCL10 with its interacting regions that bind to CARMA/CARDs (CARMA1/CARD11, CARMA2/CARD14, CARMA3/CARD10, and CARD9) and MALT1 are shown. Abbreviations: CARD, caspase-recruitment domain; CC, coiled-coil; MAGUK, membrane-associated guanylate kinase; S/T-rich, serine/threonine-rich region; DD, death domain; Ig, immunoglobulin-like domain; Para, paracaspase domain; BCL10, B-cell lymphoma/leukemia 10; MALT, mucosa-associated lymphoid tissue.

The BCL10 gene has been originally cloned from the 1p22 break-point of the chromosomal translocation t(1;14)(p22;q32) associated with mucosa-associated lymphoid tissue (MALT) lymphoma (1). In parallel homology, searches and yeast-two-hybrid screens identified BCL10 (also named c-E10, CIPER, CLAP, or CARMEN) as a protein that is ubiquitously expressed in human tissues and involved in apoptosis by virtue of its N-terminal CARD (2–6). However, while demonstrating that BCL10 overexpression is poorly activating pro-apoptotic pathways, BCL10 oligomerization through the CARD was shown to strongly induce NF-κB activation (1–3). Subsequent work revealed that BCL10 connects through heterotypic CARD–CARD interaction to the CARD-containing scaffold proteins CARMA1 (CARD11), CARMA2 (CARD14), CARMA3 (CARD10), and CARD9 (Figure 1) (7–11). In addition, BCL10 interacts with the MALT1 paracaspase (also known as PCASP1) (12), which was initially discovered from a chromosomal translocation leading to the expression of the oncogenic API2-MALT1 fusion protein in MALT lymphoma (13, 14). Besides its proteolytically active paracaspase domain (15, 16), MALT1 contains a death domain (DD) and two Ig domains (Ig1/2) in the N-terminus that strongly augment NF-κB activation by binding to a region C-terminal to the BCL10 CARD (17). Thus, initial studies mainly based on overexpression indicated that Bcl10 acts as an inducer of IκB kinase (IKK)/NF-κB signaling by bridging CARD-containing scaffold proteins with the MALT1 paracaspase.

## BCL10 Mediates Adaptive and Innate Immune Activation by Various CBM Complexes

**Figure 2:**
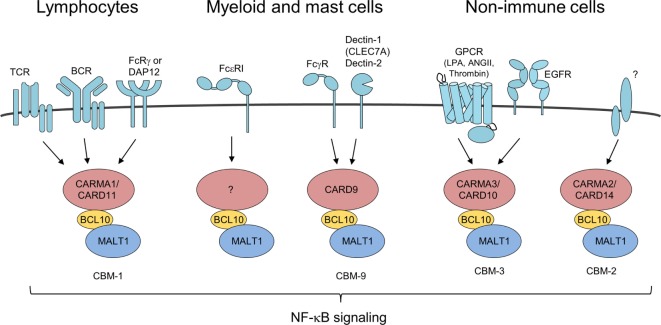
Different CARMA/caspase-recruitment domains (CARDs) connect to B-cell lymphoma/leukemia 10 (BCL10)-mucosa-associated lymphoid tissue (MALT)1 in response to distinct upstream stimuli BCL10/MALT1 complex recruitment to different CARMA/CARD proteins is dependent on activation of various receptor types forming distinct CBM complexes. All signaling pathways converge upon formation of CBM complexes leading to IκB kinase/NF-κB and JNK activation similarly.

The generation of BCL10^−/−^ mice gave insights into the physiological role of BCL10. As a result of defective neural tube closure, BCL10 deficiency leads to embryonal lethality in approximately one-third of the animals (18). Interestingly, even though the phenotype is not shared with other CARMA/CARD proteins or MALT1, failure of neural tube closure is seen in TRAF6 KO mice with a similar penetrance (19). In TRAF6 deficient animal, it has been attributed to a reduction in programmed cell death in the developing of the central nervous system, suggesting that deregulation in apoptotic processes may be responsible for embryonal defects in BCL10 KO mice. However, cells from BCL10-deficient mice displayed normal susceptibility to a number of apoptotic stimuli, indicating that BCL10 does not directly affect apoptosis signaling (18). Born BCL10^−/−^ mice are viable and reveal no gross developmental defects. Even though BCL10 promotes survival of thymocytes, it is dispensable for overall T or B cell lineage commitment (18, 20). However, more detailed immune phenotyping showed that BCL10 is required for the proper development of regulatory T cells, natural killer (NK), and NKT cells as well as marginal zone (MZ) and B1 B cells (21, 22). T and B lymphocytes from BCL10-deficient mice are defective in NF-κB signaling, fail to upregulate cytokines like IL-2, and do not proliferate in response to TCR and BCR stimulation, which leads to blunted antigen responses and severe immune deficiency (18, 22). Similar signaling defects have been observed in mice lacking CARD11 or MALT1, indicating that BCL10 together with these two proteins orchestrates antigen signaling to mount an efficient adaptive immune response (23–28). In line, antigen stimulation induces the assembly of the higher-order CBM-1 signaling complex consisting of the core subunits CARMA1/CARD11, BCL10, and MALT1 (Figure 2) (29). The CBM-1 complex is lymphocyte-specific, because CARD11 expression is largely restricted to lymphoid tissues (8, 9).

As part of the CBM-1 complex, BCL10 is also required for efficient glutamine uptake by the amino acid transporter ASCT2 and an optimal activation of the protein kinase mammalian target of rapamycin (mTOR) in CD4 T cells after TCR/CD28 co-stimulation (30). In this pathway, the CBM-1 complex regulates the differentiation of naive CD4 T cells into Th1 or Th17 cells independent of its role in canonical IKK/NF-κB signaling. By utilizing shRNA or CARD11 KO Jurkat T cells, a second study confirmed the role of CARD11 and MALT1 in regulating TCR/CD28-induced mTOR activation, but BCL10 appeared to be dispensable for the pathway, which may be due to inefficient knockdown (31). Notably, the study demonstrated that MALT1 protease activity is required for mTOR activation in T cells. The exact pathway connecting CBM-1 and mTOR remains elusive, but the data point to a critical role of MALT1 substrate cleavage independent of IKK activation.

Besides its role in the lymphoid CBM-1 complex, BCL10 controls a number of other immune and pro-inflammatory pathways *via* its recruitment to various CARMA/CARD–BCL10–MALT1 complexes (Figure 2). In NK cells, BCL10 also in conjunction with CARD11 triggers NF-κB signaling and cytokine production upon antibody binding to FcRγ (32). By coupling Fcε receptor I (FcεRI) stimulation to NF-κB activation and secretion of the cytokines IL-6 and TNFα, BCL10 contributes to IgE-associated chronic allergic diseases, but the CARMA/CARD scaffold channeling FcεRI responses is unknown (33, 34). Chen et al. also reported on a moderate impairment of mast cell degranulation in BCL10^−/−^ cells, which was not seen in the previous study and these differences may be attributed to the different markers used for detecting degranulation. In myeloid cell the alternative CARD9–BCL10–MALT1 (CBM-9) signalosome mediates FcγR stimulation as well as anti-fungal responses from C-type lectin receptors like Dectin-1 (CLEC7A) and Dectin-2 (32, 35, 36). In addition, BCL10 acts as a core component of the CARMA3/CARD10–BCL10–MALT1 (CBM-3) signaling complex that links G-protein-coupled receptor (GPCR) activated by lysophosphatidic acid, angiotensin II, or thrombin that induce pro-inflammatory gene expression also in non-immune cells (37–43). Moreover, the CBM-3 complex is crucial for NF-κB activation and tumor progression after epidermal growth factor stimulation in breast cancer cells (44). Aside from extracellular ligands, excess of intracellular free fatty acids resulting from high fat diet increases cellular diacylglycerol to promote atypical CARMA3/CARD10–BCL10-dependent, but MALT1-independent NF-κB activation in hepatocytes and thereby BCL10 deficiency contributes to insulin resistance (45). How such a CB-3 subcomplex triggers downstream signaling needs to be resolved. Finally, recent data demonstrated psoriasis associated mutations in CARMA2 (CARD14) are promoting formation of the CARMA2/CARD14–BCL10–MALT1 (CBM-2) complex to induce chronic inflammation in keratinocytes (46, 47). However, it is presently unclear what physiological stimuli and upstream signaling pathways govern CBM-2 complex activation. Taken together, BCL10 is recruited to different CARMA/CARD scaffolds to channel TCR/BCR, FcR, and GPCR signaling to induce immune and pro-inflammatory gene activation.

The crucial role of BCL10 for adaptive and innate immune activation was also observed in a human patient carrying a homozygous splice-site mutation that caused complete absence of BCL10 mRNA and protein (48). The autosomal–recessive BCL10 deficiency in humans caused combined immunodeficiency that largely mirrored the phenotype of BCL10^−/−^ mice with respect to lymphocyte signaling and activation. Profound deficits in memory B and T cells were observed in the patient that were much more prominent than in BCL10^−/−^ mice, most likely because the animals are housed in a largely sterile environment.

Conflicting results have been obtained regarding the requirement of BCL10 for innate toll-like receptor (TLR) pathways. Whereas MZ B cells, but not follicular (FO) B cells, fail to activate NF-κB in response to the TLR4 ligand lipopolysaccharides (22), the role of BCL10 for TLR signaling in myeloid cells is less clear. Hara et al. reported blunted TLR2, TLR4, TLR7, and TLR9 responses in CARD9 and BCL10-deficient dendritic cells (DCs), but Gross et al. failed to see any effects on TLR signaling in bone marrow-derived DCs (36, 49). Biochemical analyses showed that BCL10 can deliver full TLR4 responses in epithelial cells (50), suggesting that the effect of BCL10 may differ depending on the cell type and the exact stimulatory conditions. Interestingly, TLR and Dectin-1 signaling pathways are normal in human myeloid cells from a BCL10-deficient patient, but signaling is impaired in epithelial cells and fibroblast from the same patient, supporting the notion that the requirement for BCL10 in some of these pathways may be cell type specific (48). However, the exact mechanism how BCL10 influences MYD88-dependent innate responses and whether this is directly associated with assembly of a distinct CBM complex or results from more indirect effects is unclear.

## Oncogenic BCR Signaling in Lymphomas Relies on BCL10

Originally, BCL10 was cloned from the chromosomal translocation t(1;14)(p22;q32) found in MALT lymphoma (1). The translocation juxtaposes the intact BCL10 gene next to the IGH enhancer, which promotes overexpression and nuclear translocation of BCL10 (1, 51). In line, heterologous expression of BCL10 in B cells of mice induces NF-κB and B cell hyperplasia (52). However, translocation and mutations in BCL10 are rare events and genetic alterations may not have a strong contribution for lymphomagenesis (53). Still, as part of the CARD11-containing CBM-1 complex, BCL10 is an essential factor for survival of the activated B cell type of diffuse large B cell lymphoma (ABC DLBCL) that are addicted to chronic BCR signaling (54). In ABC DLBCL cells, BCL10 channels oncogenic signaling driven by somatic mutations in CARD11 or the BCR adaptors CD79A/B to MALT1 protease activation and anti-apoptotic NF-κB [for review, see Ref. (55)]. Thus, even though somatic BCL10 mutations are rare, uncontrolled BCL10 activation in B cells contributes to lymphomagenesis.

## BCL10 Filaments—Higher-Order Assembly to Promote and Amplify TCR Signaling

Immunofluorescence microscopy after overexpression showed that BCL10 exhibits a clear pattern of distinct inter-connected cytoplasmic filaments (56). Mutations in the CARD of BCL10 abrogated these BCL10 assemblies and NF-κB activation, suggesting that cellular filament formation is an essential step for BCL10 activation. Subsequent work in T cells demonstrated that BCL10 is enriched in cytoplasmic structures called POLKADOTS (punctate and oligomeric killing or activating domains transducing signals) upon TCR stimulation (57, 58). The BCL10 foci are highly dynamic platforms for information exchange between BCL10 and CBM-1 signalosome binding partners such as CARD11 and TRAF6 to foster TCR-mediated signaling towards NF-κB.

**Figure 3:**
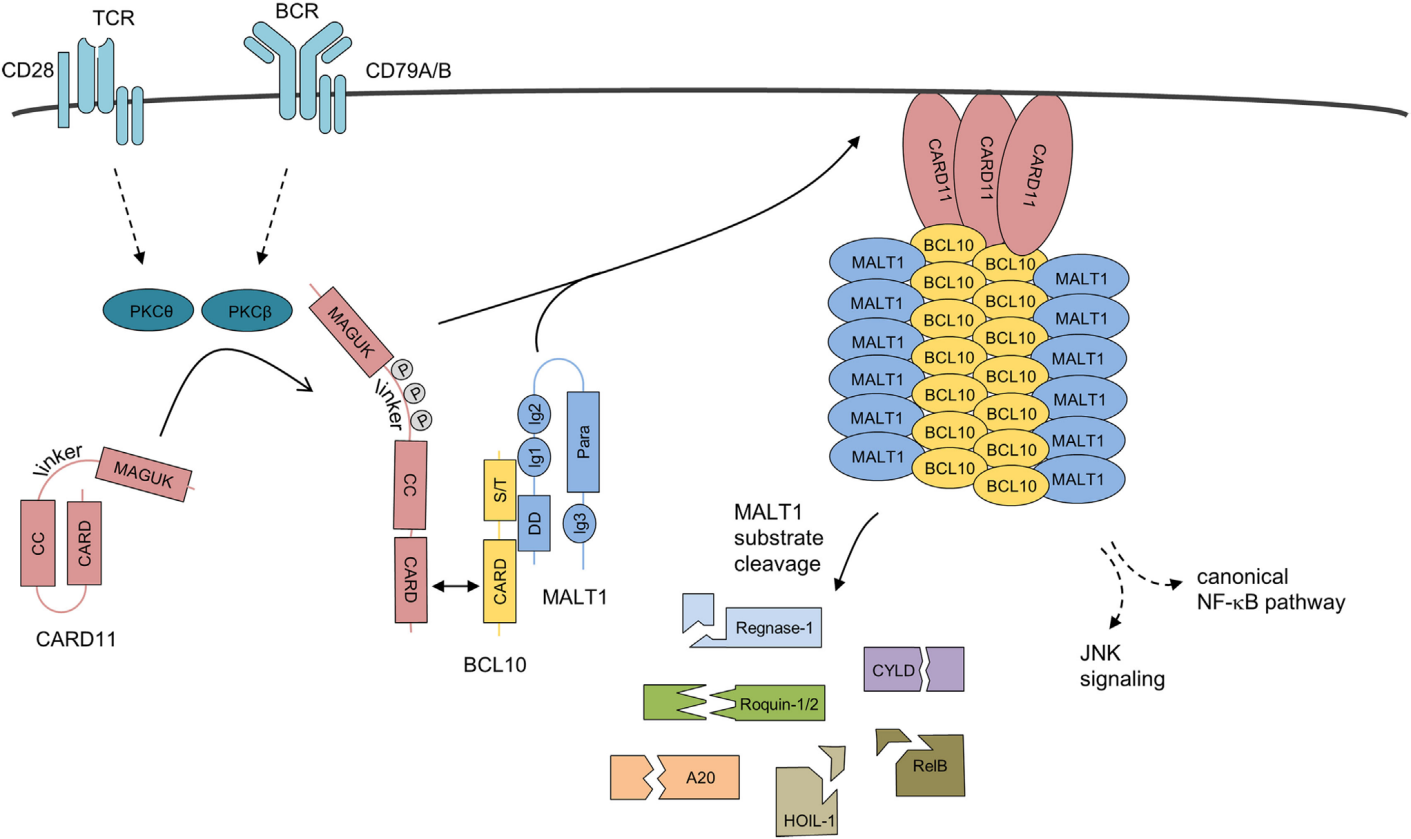
Assembly mechanism of the higher-order CBM-1 complex. Upon antigen receptor ligation on lymphocytes, CARD11 is released from its inactive state by phosphorylation and initial homotypic CARD–CARD oligomerization. CARD11 clusters at the cell membrane, induce the recruitment of pre-assembled BCL10-MALT1 complexes, and serve as a molecular seed for the formation of BCL10 filaments. BCL10–MALT1 filaments provoke NF-κB and JNK activation. In parallel, MALT1 protease is activated, which catalyzes cleavage of various cellular substrates. Abbreviations: CARD, caspase-recruitment domain; CC, coiled-coil; MAGUK, membrane-associated guanylate kinase; S/T, Ser/Thr-rich region; DD, death domain; Ig, immunoglobulin-like domain; Para, paracaspase domain; BCL10, B-cell lymphoma/leukemia 10; MALT, mucosa-associated lymphoid tissue.

The architecture and assembly of the BCL10 filaments was uncovered by cryo-electron microscopy (Figure 3) (59, 60). *In vitro* BCL10 assembles into helical filaments with a left-handed symmetry and three to four BCL10 subunits per helical turn. The hollow helix is formed by three types of homotypic CARD–CARD interactions and point mutations in the interfaces abolish BCL10 filament formation as well as MALT1 and NF-κB activation in T cells, highlighting the importance for filament assembly *in vivo* (60). Indeed, heterotypic CARD–CARD contacts between CARD11 and BCL10 are selectively localizing to the tip of the BCL10 filaments, and thereby CARD11 acts as the molecular seed that nucleates BCL10 filaments (59, 60). In the first step, the positively charged CARD11 surface recruits the negatively charged BCL10 surface (60, 61). Subsequently, the CARD11-bound BCL10 exposes its basic CARD surface to engage BCL10 monomers again *via* their negatively charged surfaces ensuring a unidirectional assembly of the growing filaments. The unidirectional growth was confirmed by time-lapse confocal microscopy (59). In line with this model, CARD11 significantly decreases the lag time for BCL10 nucleation, but had no impact on the speed of the extension of the BCL10 filaments. Interestingly, the star-shaped networks indicate that multiple filaments can emanate from one seed, which may rely on the oligomeric status of CARD11 (59, 60).

To foster downstream signaling, the BCL10 core is decorated by MALT1 and TRAF6 in an all-or-none fashion, leading to filaments with a strongly enlarged diameter (59). However, the resolution of BCL10–MALT1–TRAF6 filaments is not yet sufficient to characterize the exact binding surfaces that facilitate these interactions in the context of the CBM holo-complex. Regarding the BCL10–MALT1 interface either deletions in the DD or Ig1/2 domains in MALT1 or mutations and deletions in the CARD and Ser/Thr-rich C-terminus of BCL10 are impairing the interaction (17, 62, 63). Thus, the efficient BCL10–MALT1 binding is most likely relying on the overall conformation of both proteins. The BCL10–MALT1 filaments appear to present a dynamic surface for the clustering of signaling components like the E3 ligase TRAF6 that binds to the C-terminus of MALT1 and catalyzes MALT1 poly-ubiquitination to recruit the IKK complex and induce NF-κB activation (29, 64, 65). It needs to be noted that TRAF6 deficiency does not hamper TCR/CD28-induced NF-κB signaling in murine CD4 T cells (66). Nevertheless, mutation of the TRAF6 binding motifs in MALT1 abolish NF-κB activation in Jurkat T cells as well as murine CD4 T cells (67), strongly arguing that other E3 ligase(s) can compensate for the loss of TRAF6 in primary T cells. Further work is needed to elicit the exact architecture, the assembly, and the dynamics of BCL10 filaments and the CBM holo-complex, to understand mechanistically how different players are integrated to shape activation and termination of immune signaling.

## Control of CBM Complex Dynamics by Post-Translational Regulation of BCL10

**Figure 4:**
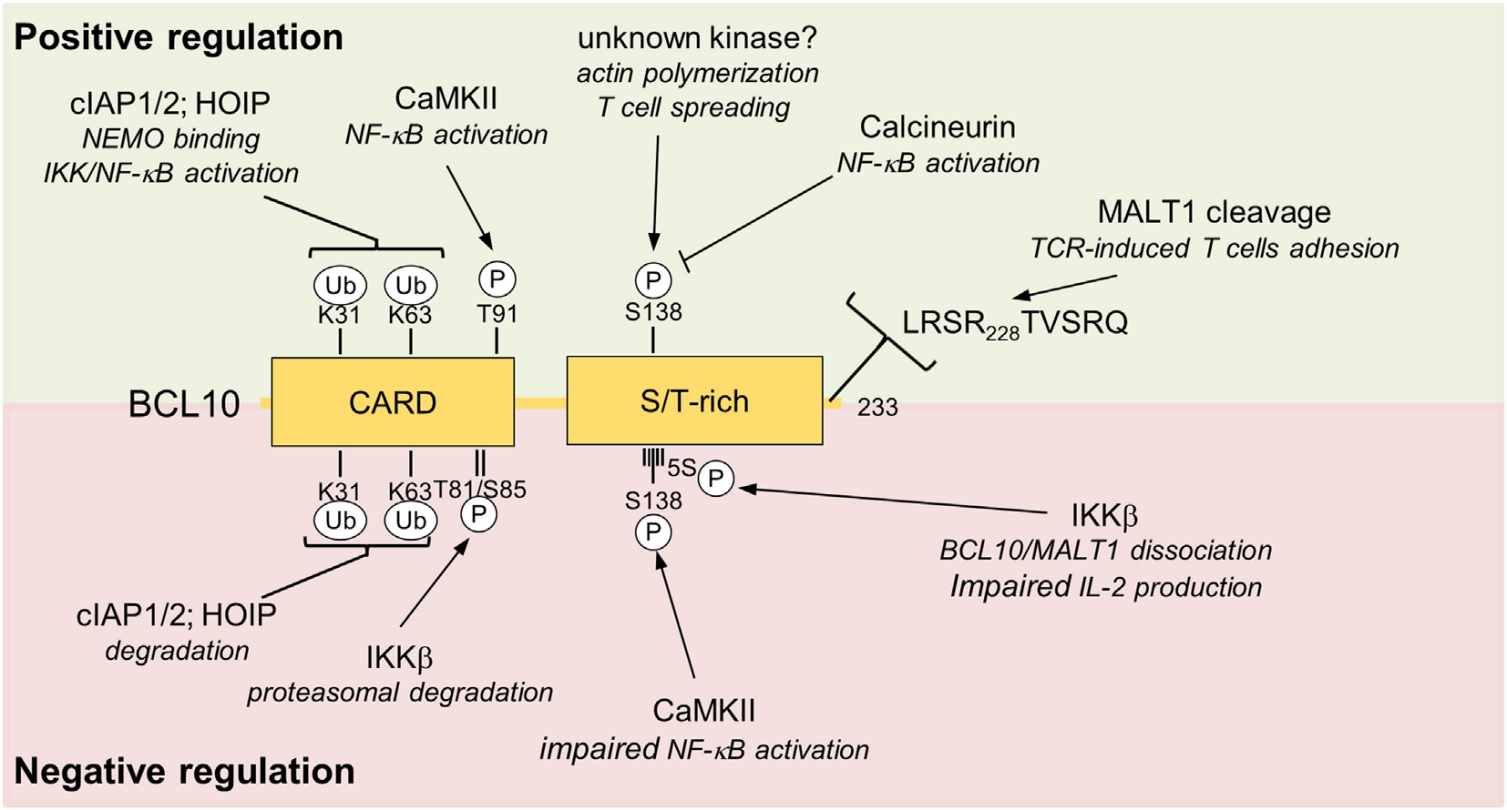
Positive and negative regulations of BCL10. Positive and negative regulating post-translational modification sites (phosphorylation and ubiquitination) and MALT1 cleavage site are depicted. Upper box highlighted in green shows modifications of BCL10 associate with positive impact on T cell activation. The red lower box summarizes negative regulatory effects that impinge on BCL10. Abbreviations: CARD, caspase-recruitment domain; S/T-rich, serine/threonine-rich region; BCL10, B-cell lymphoma/leukemia 10; MALT, mucosa-associated lymphoid tissue.

Initial phosphorylations of CARMA/CARD scaffolds have been demonstrated to control the recruitment of pre-assembled BCL10–MALT1 and thus CBM complex assembly after stimulation (68, 69). Subsequently, BCL10 is regulated by a variety of post-translational processes including phosphorylation, ubiquitination, complete degradation, and C-terminal cleavage (Figure 4).

### BCL10 Phosphorylation Balances TCR Signaling

B-cell lymphoma/leukemia 10 is prone to extensive phosphorylations upon T cell activation, which initially was suggested to foster NF-κB signaling (70, 71). Calmodulin kinase II (CaMKII) was found to associate with BCL10 and promote NF-κB signaling by phosphorylating threonine 91 in the CARD domain (72). Phospho-defective or -mimetic mutations were not interfering with CARD11 association, but it needs to be determined if threonine 91 is accessible in the context of the BCL10 filament and if phosphorylation affects filament structure and/or downstream signaling. Recently, GSK3β was also identified as a BCL10 kinase that augments CBM-1 complex formation and NF-κB activation in T cells, but the exact phosphorylation sites still need to be mapped and their functional relevance needs to be determined (73).

Other work demonstrated that BCL10 phosphorylations are largely counteracting CBM-1 signaling, and thereby exert negative regulatory roles on T cell activation (63, 74–76). The protein kinases IKKβ can catalyze BCL10 phosphorylation at multiple serines (134, 136, 138, 141, and 144) in the C-terminus and phospho-defective mutations lead to increased signaling and IL-2 production, indicating that IKKβ besides its positive role for NF-κB signaling can also put a brake on T cell activation (63). Especially phosphorylation of serine 138, which is also modified by CaMKII, is decreasing NF-κB activation (74, 76). Interestingly, C-terminal phosphorylations impair binding of BCL10 to MALT1, suggesting BCL10 phosphorylation balances signaling by structurally remodeling the CBM-1 complex (63). Phosphorylation at serine 138 was also suggested to enhance BCL10 turnover by a proteasome-independent mechanism (76), but an effect of this phosphorylation on BCL10 stability was not confirmed in other studies (77–79). The discovery of Calcineurin as a BCL10 phosphatase in T cells lends strong support to the negative control of BCL10 by protein kinases (78, 80). Inhibition by cyclosporine A or knockdown of Calcineurin augments IKKβ- or CaMKII-catalyzed BCL10 phosphorylation and persistent phosphorylation of serine 138 after TCR stimulation impedes IKK/NF-κB signaling, providing proof for the counterbalancing effect of BCL10 phosphorylation (78, 80).

Phosphorylation of BCL10 at serine 138 has also been implicated in TCR-induced actin polymerization and cell spreading of T cells (79). Furthermore, FcγR-mediated actin polymerization and phagocytosis in macrophages was impaired by BCL10 knockdown. Interestingly, this process was independent of CARD11 and MALT1, pointing to a role of BCL10 outside the CBM complex (79). Subsequent work confirmed the NF-κB-independent role of BCL10 in actin and membrane remodeling downstream of FcR in human macrophages by complexing with clathrin adaptors and recruiting the phosphatase OCRL that controls phosphatidylinositol-4,5-bisphosphate and F-actin turnover (81). The protein kinases controlling actin polymerization through serine 138 phosphorylation are unknown, but it is tempting to speculate that with IKKβ and CaMKII the same kinases that counteract NF-κB signaling by phosphorylating in this region, may also exert positive effects on actin polymerization. Certainly, further work is needed to understand the physiological relevance of BCL10-dependent actin polymerization for innate and adaptive immune responses.

Besides phosphorylation in the C-terminal Ser/Thr-rich region, IKKβ was shown to trigger phosphorylation of threonine 81 and serine 85 in the CARD of BCL10, which mediates the recruitment of Cullin1-βTRCP1 E3 ligase complex and proteasomal degradation of BCL10 (75). Since the accessibility of these residues in the BCL10 filaments is unclear and the majority of the BCL10 aggregates are removed by selective autophagy (see below) (82), IKKβ-dependent degradation may influence a distinct pool of BCL10 not attached to the CBM complex. Thus, even though the negative regulatory function of BCL10 phosphorylation on TCR activation has been confirmed in different studies, the exact regulations and mechanisms are still not completely resolved.

### Balancing of BCL10 Activation and Degradation by Ubiquitination

Upon stimulation of antigen receptor signaling pathways in T and B cells, BCL10 is heavily modified by poly-ubiquitin chains, which have been associated with triggering downstream signaling pathways as well as BCL10 degradation (83–86). Indeed, BCL10 degradation and removal after T cell co-stimulation is required for post-inductive termination of CBM complex signaling (85). Mechanistically, TCR engagement promotes K63-linked ubiquitination of BCL10 leading to p62/Sequestosome-1-dependent autophagy and subsequent lysosomal degradation (82, 85). Also proteasomal BCL10 degradation was suggested to occur upon phosphorylation (75), but the efficient removal of the higher-order BCL10 filaments that form cellular clusters after lymphocyte stimulation can most likely only be achieved by selective autophagy and the delivery to lysosomal vesicles (57, 60, 82). Of note, MALT1 is spared from selective autophagy despite its constitutive association with BCL10 (82). How MALT1 is secluded from lysosomal destruction is not understood, but it may involve dissociation from hyper-phosphorylated BCL10 (63).

As mentioned earlier, ubiquitination of BCL10 is also required for T cell activation (86). Lysines 31 and 63 in the CARD domain serve as attachment sites of poly-ubiquitin chains, which promote the efficient recruitment of the IKK regulatory subunit NEMO (IKKγ) to BCL10 and IKK/NF-κB signaling (86). Thus, in conjunction with MALT1 ubiquitination, ubiquitin chains attached to BCL10 are mediating optimal IKK/NF-κB signaling after assembly of the CBM-1 complex in T cells (29, 86). Adding to the complexity, mutation of lysines 31 and 63 also leads to the stabilization of BCL10 in T cells after stimulation, suggesting that the same lysine residues are required for activation and termination of CBM-1 signaling (86).

A number of E3 ligases have been suggested to catalyze BCL10 poly-ubiquitination. Upon overexpression HECT E3 ligases NEDD4 or ITCH counteract TCR/CD28-induced NF-κB activation by promoting BCL10 poly-ubiquitination and degradation (84, 85). Indeed, ITCH deficiency is enhancing BCL10 turnover in primary T but not B cells and ITCH or NEDD4 are recruited to BCL10 *via* the protein kinase TAK1 independent of its kinase activity (77). TAK1 overexpression also decreases BCL10 amounts in ABC DLBCL cell lines that are characterized by chronic BCR signaling. In these tumor cells, kinase inactive TAK1 is responsible for BCL10 degradation and NF-κB inactivation. At the same time, TAK1 activity induces JNK signaling and both processes are required to induce cell death, and thereby counterselect BCR-addicted lymphoma cells (77).

Also cIAP2 induces BCL10 degradation, but inhibition of cIAP1/2 by antagonist did not affect BCL10 turnover in B cells (77, 84). In fact, in ABC DLBCL tumor cells cIAP1/2 antagonists interfere with NF-κB-dependent pro-survival signaling by preventing the conjugation of K63-linked poly-ubiquitin chains on lysine 31/63 of BCL10 (87). However, since both lysine residues are also involved in degradation of BCL10, it is presently unclear how cIAP-dependent processes are balanced. In addition, most likely through K63-linked auto-ubiquitination, cIAPs recruit the linear ubiquitin chain assembly complex (LUBAC) consisting of HOIP (RNF31), HOIL-1 (RBCK1), and SHARPIN to the CBM-1 complex in ABC DLBCL cells (87, 88). Linear methionine (Met)-1 linked ubiquitination of NEMO by the LUBAC was initially found to be critical for TNFα-dependent NF-κB signaling and several studies demonstrated that LUBAC also controls NF-κB activation and survival of ABC DLBCL cells (88, 89). Furthermore, HOIP catalyzes linear ubiquitination of BCL10 in chronically activated ABC DLBCL as well as T and B cells in response to TCR and BCR stimulation, respectively (87, 90, 91). NF-κB activation is reduced after TCR/BCR cross-linking in HOIP-deficient T and B cells (89–91) and at least partially overlapping attachment sites on BCL10 have been mapped for Met1- and K63-linked ubiquitination (K14, K31, and K63 in BCL10) (91). However, neither HOIP deficiency nor mutation of BCL10 attachment completely abolished NF-κB activation, suggesting that Met-1 or K63 ubiquitination of BCL10 may not be essential but modulate CBM-1 activity. In fact, one study suggested that LUBAC may have a scaffolding function that facilitates NF-κB activation independent of HOIP catalytic activity (89).

A number of BCL10 E3 ligases have been identified, but much less is known about deubiquitinating enzymes (DUBs) counteracting BCL10 ubiquitination. *In vitro* the Met-1-selective DUB OTULIN can remove linear ubiquitin chains from BCL10 (91), but its role *in vivo* is unclear as T and B lymphocytes from conditional OTULIN KO mice are not chronically activated (92). BCL10 ubiquitination and degradation is enhanced in Jurkat T cells lacking the components of the COP9-signalosome (CSN), a multimeric complex with isopeptidase activity (93). Since the CSN is primarily influencing ubiquitin conjugation by deneddylating Cullin-RING E3 ligases complexes, the CSN may affect BCL10 stability indirectly. Finally, the ubiquitin-specific protease 9X (USP9X) interacts with BCL10 in activated T cells and silencing of USP9X augments BCL10 ubiquitination and impairs NF-κB signaling in T cells (94). Even though USP9X counteracts the assembly of K48-linked ubiquitin chains on BCL10, this does not lead to decreased BCL10 stability. At present, it is unclear how the regulation by USP9X is connected to the previously described E3 ligases and ubiquitination events, but augmented K48-ubiquitination may compete for the attachment of activating ubiquitin chains with other topologies to BCL10 and thus may promote a dysfunctional CBM-1 response.

Taken together, BCL10 acts as a platform that integrates many components of the ubiquitin system, which control BCL10 activity and stability in lymphocytes and lymphoma cells. Even further BCL10 E3 ligases, like mind bomb-2 or RNF8/RNF168 have been identified, but their role in BCL10-dependent immune signaling is unclear (95, 96). The exact order of events and the contribution of individual ubiquitin ligases and DUBs during the activation and termination phases of immune cell signaling are still to be explored.

### BCL10 Cleavage by the MALT1 Protease

Upon activation, MALT1 protease cleaves a number of substrates with key functions in signaling as well as transcriptional and post-transcriptional regulation (Figure 3) (97). MALT1 protease also catalyzes cleavage of BCL10 at arginine 228 removing the last five amino acids from the C-terminus of BCL10 after T cell stimulation (16). C-terminal truncation of BCL10 is also visible in ABC DLBCL cells that are characterized by constitutive MALT1 activity (98, 99). Even though MALT1 protease activity enhances expression of NF-κB target genes like IL-2 in T cells, cleavage of BCL10 is not critical in this process. However, MALT1-dependent cleavage of BCL10 was required for integrin-dependent adhesion of T cells to fibronectin, which is important for the efficient contact of T cells to antigen-presenting cells (16). Interestingly, also thrombin-induced monocyte adhesion is dependent on endothelial BCL10 and thus an intact CBM-3 signalosome, but a putative involvement of the MALT1 protease and BCL10 in this process is unknown (37). Since CBM-dependent NF-κB regulation upregulates several genes controlling adhesion (e.g., ICAM-1, VCAM), it needs to be sorted out whether differences in adhesion may result from differential target gene expression after expression of cleavage defective BCL10 or MALT1 inhibition. To elucidate the physiological relevance of MALT1-catalyzed BCL10 cleavage, a mouse model expressing a cleavage resistant BCL10 R228A variant would be required.

## Conclusion and Outlook

Since its discovery an enormous amount of data has been gathered that support the key function of BCL10 as the linker of all CBM complexes. By connecting to the scaffold proteins CARD11, CAR14, CARD10, and CARD9, BCL10 filaments channel antigenic stimulation in lymphocytes and distinct innate and inflammatory stimuli in many other cells to the NF-κB and JNK signaling pathways. BCL10 is the essential adaptor to initiate downstream signaling as well as MALT1 protease activity and substrate cleavage, which is balancing immune cell activation. Thus, BCL10 is mediating the known effects of the CARD scaffolds and acts as the bridge of all CARMA/CARDs involved in immune signaling. BCL10 is prone to numerous post-translational modifications, processing, and degradation, which control binding of interaction partners, stability and activity, highlighting that the role of BCL10 goes far beyond its function as the CBM linker. However, despite first proteomic analyses revealing BCL10 interactions and modifications in activated B cells (90), we are lacking a clear picture about the BCL10 regulation during immune cell activation. Global analyses are needed that cover the dynamics of BCL10 modifications, interactions, localization, and degradation, to elucidate the exact order of events. Moreover, we are still lacking a clear understanding how BCL10 filaments and thus the CBM holo-complexes are assembled und how this highly dynamic signalosome channels to downstream signaling pathways. More structural and functional analyses as well as high resolution imaging are required to elucidate these processes. Several studies demonstrated functions of BCL10 in actin polymerization, phagocytosis, cell adhesion, DNA damage response, fatty acid signaling, or neural tube closure, which are presumably independent of known CARMA/CARDs and/or MALT1 (18, 45, 79, 100). It will be very interesting to explore how and to what extend BCL10 engages with other partners and functions outside the CBM complexes. Overall, in depth analyses are needed to evaluate how this important immune cell machinery can be exploited therapeutically to modulate immune responses for the treatment of autoimmune diseases, chronic or acute inflammation, and hematologic malignancies.

## Author Contributions

TG, TS, and DK designed and wrote the review.

## Conflict of Interest Statement

The authors declare that the research was conducted in the absence of any commercial or financial relationships that could be construed as a potential conflict of interest.
